# Efficacy of the cognitive functional therapy (CFT) in patients with chronic nonspecific low back pain: a study protocol for a randomized sham-controlled trial

**DOI:** 10.1186/s13063-022-06466-8

**Published:** 2022-07-04

**Authors:** Mariana Romano de Lira, Ney Armando de Mello Meziat-Filho, Gabriela Zuelli Martins Silva, Thaís Cristina Chaves

**Affiliations:** 1 Postgraduate Program in Rehabilitation and Functional Performance, Ribeirão Preto Medical School, University of São Paulo, Avenida Bandeirantes 3900, Ribeirão Preto - SP, CEP:14049-900 Brazil; 2 Postgraduate Program in Rehabilitation Sciences, Augusto Motta University Center – UNISUAM, Rio de Janeiro, Brazil; 3 Department of Physical Therapy, Federal University of São Carlos, São Carlos, São Paulo, Brazil; 4 Laboratory of Research on Movement and Pain (LabMovePain), https://labmovdor.wordpress.com/

**Keywords:** Cognitive functional therapy, Low back pain, Pain management, Placebo effect and protocols

## Abstract

**Background:**

Chronic low back pain is a public health problem, and there is strong evidence that it is associated with a complex interaction of biopsychosocial factors. Cognitive functional therapy (CFT) is a promising new intervention that deals with potentially modifiable multidimensional aspects of pain (e.g., provocative cognitive, movement, and lifestyle behaviors).

**Methods:**

To investigate the efficacy of CFT compared with a sham intervention for pain intensity and disability post-intervention (immediately after the last session) in patients with non-specific chronic low back pain (CLBP). This study is a randomized controlled trial in which 152 (18–60 years old) patients with CLBP will be enrolled. The patients will be randomly allocated to receive (1) CFT intervention or (2) sham intervention. The experimental group will receive individualized CFT in a pragmatic manner (5 to 7 sessions) based on the clinical progression of the participants. The sham group will attend six sessions: consisting of 30 min of photobiomodulation using a detuned device and more than 15 min of talking about neutral topics. Patients from both groups also will receive an educational booklet (for ethical reasons). Participants will be assessed pre and post-intervention, 3 months, and 6 months after randomization. The primary outcomes will be pain intensity and disability post-intervention. The secondary outcomes will be: pain intensity and disability at 3- and 6-month follow-up, as well as self-efficacy, global perceived effect of improvement, and functioning post-intervention, 3-, and 6-month follow-up. The patients and the assessor will be blinded to the treatment administered (active vs. sham).

**Statistical analysis:**

The between-group differences (effects of treatment), as well as the treatment effect for the primary and secondary outcomes, and their respective 95% confidence intervals will be calculated by constructing linear mixed models.

**Discussion:**

To the best of our knowledge, the current study will be the first to compare CFT vs. sham intervention. Sham-controlled RCTs may help to understand the influence of non-specific factors on treatment outcomes. Considering complex interventions as CFT, it is imperative to understand the impact of contextual factors on outcomes.

**Trial registration:**

ClinicalTrials.gov NCT04518891. First Posted: August 19, 2020.

**Supplementary Information:**

The online version contains supplementary material available at 10.1186/s13063-022-06466-8.

## Background

Low back pain is a challenge in terms of public health worldwide [1], due to its high prevalence [2] and socioeconomic impact [3]. It is considered the number one cause of disability worldwide [4]. For the management of non-specific chronic low back pain (CLBP), assessment and interventions focused on the biopsychosocial model are recommended by guidelines [5–7]. However, a large number of interventions for CLBP are not personalized to encompass specific individualized patient needs [8].

Cognitive functional therapy (CFT) is a flexible and integrated behavioral approach for individualizing the management of people with disabling CLBP [9]. Previous randomized clinical trials (RCTs) showed reductions in disability for the group submitted to CFT compared with manual therapy and exercise [10, 11] and with pain education and group-based exercise [12]. However, methodological shortcomings such as a high loss of follow-up of participants precluded confirmatory results. There are additional studies in progress [13, 14]; however, to the best of our knowledge, there is no previous study comparing CFT against a placebo treatment.

Previous findings have demonstrated that several therapies for CLBP are actually not superior or have only marginal efficacy compared with placebo for the management of CLBP in RCTs [15–20] or systematic reviews with meta-analysis [21]. In this way, it is important to conduct clinical trials comparing interventions commonly used in clinical practice against placebo ones.

The placebo could be defined as any therapy or component of therapy used for its nonspecific, psychological, or psychophysiological effect, or that is used for its presumed specific effect, but is without specific activity for the condition being treated [22]. In randomized placebo-controlled clinical trials, the placebo (term properly used in the context of pharmacological interventions) or sham (when the placebo mimics a procedure/surgery intervention) treatment arm is designed to capture and control for the nonspecific variables that can influence clinical outcomes [23]. These possible confounding could be regression to the mean, spontaneous remission, changes due to the natural course of the disease, Hawthorne effect, and placebo effects. In addition, the placebo control in a randomized trial enables the blinding of the patients and/or the investigators which facilitates outcome blinding [21, 24].

One can argue that for complex interventions such as CFT, the comparison with a sham procedure is not suitable. Consequently, the challenge is to deliver the same amount of fake therapeutic ingredients and patient-therapist interactions, which could be better called as a sham intervention. In such a scenario, it is compelling the importance of controlling for the intervention fidelity - the extent to which a behavioral intervention (active or sham) was designed, implemented, and received as intended [25, 26].

In this way, the aim of this study is to investigate the efficacy of CFT compared with a sham intervention (photobiomodulation using a detuned device + talking about neutral topics), for pain intensity and disability post-intervention in patients with CLBP. The secondary aims will be to investigate the effect of CFT for pain intensity and disability at 3- and 6-month follow-up, as well as the effect of the active treatment on self-efficacy, global perceived effect of improvement and functioning post-intervention and at 3- and 6-month follow-up.

## Methods

### Ethical considerations and protocol registration

The study was submitted to and approved by the ethics committee for research involving human subjects of the Ribeirão Preto Medical School (Ethics Committee Board from Centro Saúde Escola Cuaibá) of the University of São Paulo (CAAE: 30367320.4.0000.5414.). The study was registered prospectively on Clinical Trials (NCT04518891). The blinded assessor will clarify all details of the research and will obtain informed consent from all volunteers prior to allocation. No significant adverse reactions are anticipated in the study, but these will be monitored. The intervention is planned to be discontinued if there is withdrawal of participant consent.

### Setting and participants

#### Study participants and eligibility criteria

This study will be a superiority RCT, sham-controlled, prospectively registered, two-arm with a blinded assessor. The sample will be comprised of 152 participants (both genders) with non-specific CLBP who will be referred to the physiotherapy outpatient clinic from Ribeirão Preto Medical School – University of São Paulo (Brazil), as well as community volunteers recruited through invitations announced by social media (Instagram and Facebook). Participants who met the following criteria will be considered eligible for the study: (1) aged between 18 and 60 years; (2) current episode of non-specific CLBP for at least 3 months of duration diagnosed by a general practitioner (different qualified physicians will be in charge of the diagnosis), that is located between T12 and the gluteal folds; (3) pain intensity equal to or greater than three on a numerical pain rating scale (NPRS); (4) score greater than 14% on the Oswestry Lumbar Disability Index [27]; and (5) fluent in Brazilian Portuguese.

Participants will be excluded due to the following conditions: (1) red flags (neoplastic diseases or tumors in the spine, inflammatory diseases, infections, and fractures); (2) serious neurological (or central and peripheral neurological) symptoms and psychiatric, rheumatologic, and cardiac diseases; (3) radiculopathy with symptoms (evidence of nerve root compromise tested by clinical neurological examination—identifying motor, reflex, or sensory abnormalities); (4) lumbar stenosis; (5) spondylolisthesis; (6) history of spinal surgeries; (7) pregnancy; (8) underwent other physical therapy treatments for low back pain or chronic pain in the last 6 months; and (9) illiterate people. Patients will be oriented to not use pain relief medications during the application of the interventions and during the one-month follow-up, they will be encouraged to register in a pain log the use of pain killers or rescue medications [28].

### Procedures, randomization, and allocation

The study will follow the recommendations described on Consolidated Standards of Reporting Trials (CONSORT) statement [29], the current report followed the Standard Protocol Items: Recommendations for Interventional Trials (SPIRIT) document [30], and the Template for Intervention Description and Replication (TIDieR) checklist for placebo and sham controls [31]. The description of interventions as recommended by TIDieR can be found in supplementary material 1.

**Fig. 1:**
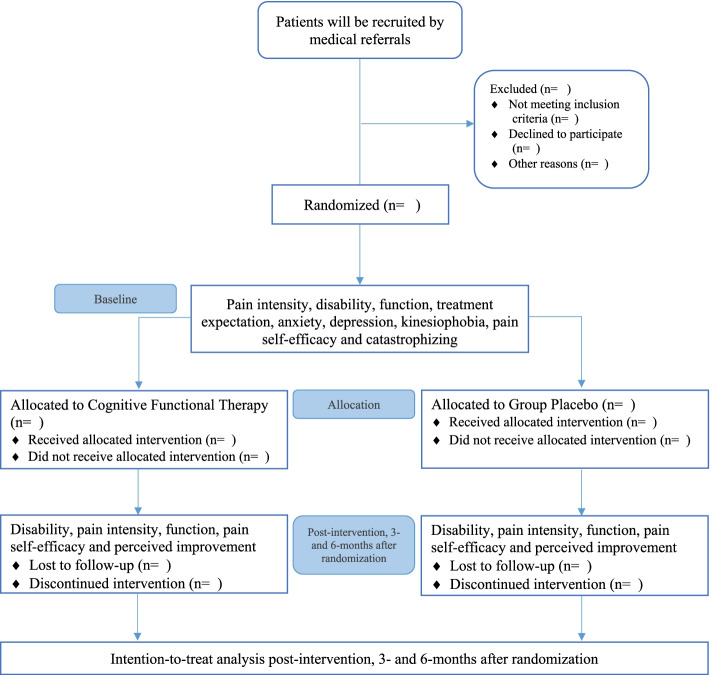
Study flow chart according to Consolidated Standards of Reporting Trials (CONSORT) statement

Patients will be informed that this study will involve a sham intervention arm (fake intervention in which an inert treatment will be provided but it still can result in positive outcomes due to its psychological effects) vs. an active treatment arm, but the nature of the sham will not be elucidated (equipment detuned). After this initial assessment, participants will be randomly assigned using block randomization by simple computerized procedures to one of the two treatment groups through the use of cards previously placed in opaque sealed envelopes: (i) CFT group or (ii) sham group. The allocation sequence will be generated by a researcher not involved in the assessment and interventions (TCC), and another research assistant will assign participants to interventions (Fig. 1). Each participant will be treated by a single physiotherapist, who will not be involved in the assessment of the patients. Participants will not have knowledge about treatment allocation. One blinded researcher regarding treatment group allocation will run the assessments pre-treatment, immediately after, and at the follow-ups.

### Outcome measures

The primary outcomes in the current study will be pain intensity [32] and low back pain-related disability [27] collected post-intervention. The secondary outcomes will be pain intensity and low back pain-related disability at 3 and 6 months after randomization, as well as self-efficacy [33], global perceived effect of improvement [32], and functioning [32] post-intervention, 3, and 6 months after randomization. In the baseline, we will administer scales to assess moderating effect: expectation [34], depression [35], anxiety [36], kinesiofobia [37], and pain catastrophizing [38]. Mini-Mental State Examination (MMSE) [39] will be administered to assess the cognitive function of the volunteers. Patients with a score lower than 24 will be excluded [40]. The questionnaires will be administered in an assisted interview mode.

### Primary outcomes

#### Pain intensity

Numeric pain rating scale (NPRS) will be used to assess pain intensity. The NPRS used in this trial will consist of numbers from 0 to 10, in which 0 represents “no pain” and 10 represents “worst pain imaginable” [32]. NPRS showed responsiveness to change, with a minimum clinically important difference (MCID) of 2 among patients with CLBP [41].

#### Low back pain-related disability

The Brazilian Portuguese version of the Oswestry Disability Index (ODI) [27] consists of 10 items, each of which has six response options. The total score will be calculated by summing up all the points, with the largest possible sum being 50. This sum will be transformed into a percentage (0 to 100). ODI showed responsiveness to change for patients with CLBP, with MCID of 10–12 points [41].

### Secondary outcomes

#### Pain Self-Efficacy Questionnaire (PSEQ)

The PSEQ has 10 items related individual’s confidence to perform a certain task which are rated on a 7-point ordinal scale (ranging from 0: “*not at all confident*” to 6: “*completely confident*”). It was adapted and validated to Brazilian Portuguese [33].

#### Global Perceived Effect of improvement with treatment (GPE)

GPE used for this trial is an 11-point scale that ranges from −5 (“vastly worse”) through 0 (“no change”) to +5 (“completely recovered”) and participants are asked: “Compared to when this episode first started, how would you describe your back these days?” [32].

#### Patient-Specific Functional Scale (PSFS)

The PSFS using the average of three items scored from 0 (unable to perform) to 10 (able to perform at preinjury level) [32]. In the PSFS patients are asked to identify up to three important activities that they are having difficulties with or are unable to perform due to their condition. In addition, the patients are asked to rate, on an 11-point scale (ranging from 0 to 10) their current level of ability associated with each activity.

### Baseline assessment

#### Standford ExpectationTreatment Scale (SETS)

The SETS is a scale with six items, three covering positive treatment-related expectations and three regarding negative treatment-related expectations [34]. The SETS is comprised of a seven-point Likert-type response scale that was chosen, varying from (1) “strongly disagree” to (7) “strongly agree.” An average of items 1, 3, and 5 yields the positive expectancy score, while an average of 2, 4, and 6 yields the negative expectancy score. This scale is under cross-cultural adaptation into Brazilian Portuguese.

#### Patient Health Questionnaire 9-item (PHQ-9)

The PHQ-9 is a nine-item questionnaire designed to screen for depression in primary care and other medical settings. They incorporate DSM-IV depression criteria with other leading major depressive symptoms into a brief self-report instrument. The standard cut-off score for screening to identify possible major depression is 10 or above. It was adapted and validated to Brazilian Portuguese [35].

#### The Generalized Anxiety Disorder 7-item scale (GAD7)

The GAD-7 [36] is a 7-item self-report measure of generalized anxiety symptoms grouped into one factor of generalized anxiety. Respondents score each item in a 4-point scale based on how often they have been bothered by the described symptoms over the last two weeks (not at all = 0; several days = 1; more than half the days = 2; nearly every day = 3). Total scores range from 0 to 21, with higher scores reflecting higher severity levels of anxiety. It was adapted and validated to Brazilian Portuguese [42].

#### The Tampa Scale for Kinesiophobia (TSK)

The Brazilian Portuguese version of TSK has 17 statements scored on 4-point scales from “strongly disagree” to “strongly agree,” yielding a total range from 17 to 68. Higher scores indicate more severe fear-avoidance beliefs. The Tampa Scale for Kinesiophobia was cross-culturally translated and validated into Brazilian Portuguese [37].

#### Pain Catastrophizing Scale (PCS)

The PCS translated and validated to Brazilian Portuguese [38] is composed of 13 items staggered on a Likert scale, ranging from 0 to 5 points. The total score is the sum of the items divided by the number of items answered. Higher scores indicated a greater presence of catastrophic thoughts. The total score of the scale could vary between 0 and 52 points [38].

### Blinding

Despite it being a sham-controlled RCT, considering the differences in the interventions administered in both study arms, it will not be possible to blind the therapist and the patients. The assessor, as well as the participants, will not be aware of the arm of the study that he/she will be allocated. However, considering the different nature of the interventions, we cannot guarantee that the patients’ blinding will work. The blinding codes will be kept at the monitoring office of research and research ethics till the end of the trial unless an emergency developed which requires unblinding. At the 6-month follow-up participants will be asked if they received the sham or active intervention, as a mean to confirm the blinding.

### Interventions

All the patients will receive a booklet with information regarding low back pain and advice on strategies of self-management [43]. The patients in the current study will be randomly allocated to receive one of two possible interventions: (1) CFT or (2) sham intervention. The same physiotherapist will deliver the interventions.

Patients in the sham intervention group will receive 6 sessions, lasting 45 min, once a week. Similarly, CFT treatment duration will vary from approximately 5 to 7 sessions [10, 12], once a week [44]. Patients will receive weekly reminders of the next sessions, as a strategy to improve adherence to intervention protocols. As a strategy to control for the treatment fidelity on both arms of the study, we will adopt the framework developed by the NIH Behavior Change Consortium (BCC) [45]. To control for the “Intervention Delivery,” the time of therapist-patient interaction, number of sessions, and the different components of the intervention administered, as well as video recordings along the trial, will be registered. Also, regular meetings to discuss the clinical cases will be performed. Furthermore, the “Intervention receipt” will be assessed in two different manners: (i) immediately post-intervention, each participant will be submitted to a manipulation check and they will be asked about the group they believe they were allocated, and (ii) patients in both groups will be invited to summarize at the beginning of each session how the treatment impacts their lives.

### Sham intervention

**Fig. 2:**
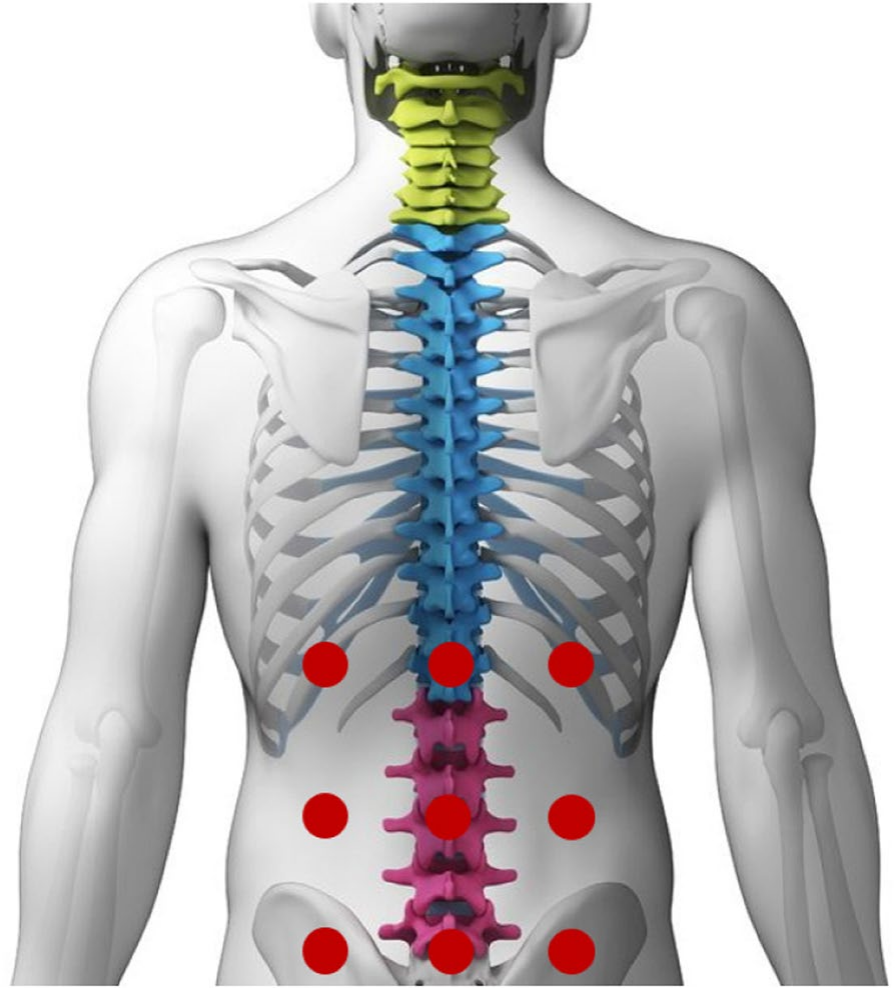
Sham photobiomodulation application sites: nine sites will be applicated on the patient’s lumbar region: three central sites on top of the spinous processes (between T11 and T12, L2 and L3, L5 and S1); in the same direction, but laterally, three sites on the left and three on the right (on the paravertebral muscles), without any emission of therapeutic dose

Patients allocated to this group will receive two interventions: sham photobiomodulation + neutral talking. Patients will be treated with detuned photobiomodulation device (904Nm Ibramed Infrared – no-visible beam), without any emission of therapeutic dose (0J). The points for applying fake stimulation are described in the Fig. 2. Three minutes of fake stimulation will be administered, summing up to 27 min [19].

The photobiomodulation will be adopted in the current study because of the absence of sensory perceptions during the application. The device will be used with the internal cables disconnected, however, it will be possible to handle it and adjust doses and alarms as if they were simulating a real clinical situation as well as to increase the credibility. Sham intervention has been used in previous trials with patients with CLBP [15, 16]. A physiotherapist with 5 years of clinical experience will administer this intervention.

In addition, a neutral talking control therapy of at least 15 min will be provided to patients in each session [46]. Maladaptive beliefs will not be challenged; however, the therapists will be trained to show interest and warmth, empathy, and encouraging participants to discuss neutral topics such as hobbies, sports, and current affairs. No advice or problem solving will be given, and any attempt to talk about emotional issues will be kindly discouraged and the talking will be redirected to neutral tropics. For example, if the patient says, “I guess I’ll never play soccer again because of my back pain”, the therapist will be trained to ask, “Do you like soccer? Did you watch the game on TV last week?”

After finishing the 6-month follow-up, all the participants will be invited to receive the CFT intervention.

### Cognitive Functional Therapy (CFT) Group

The specific training for the CFT intervention involved the following: (1) submit the treating physiotherapist with a training of at least 106 h of CFT by an experienced CFT tutor (NAMMF) and (2) videos of the physiotherapist (MRL) administering the CFT approach will be analyzed by a certified physiotherapist. The physiotherapist responsible to deliver CFT has a clinical experience of 5 years.

CFT is underpinned by a strong therapeutic alliance, active listening, motivational interviewing style (open, non-judgmental, reflective), providing validation, and using strategies to establish rapport. A customized progressive self-management program will be provided tailored to the individual’s valued goals, directed at changing cognitive, movement, and lifestyle behaviors considered provocative or aggravating of their condition.

The CFT intervention has three broad components [44]:

1. *Making sense of pain*: a reflective process that combines the person’s own narrative (interview) and experience to develop a customized relevant, multidimensional understanding of pain for the patient. In this process, maladaptive beliefs related to a vicious cycle of persistent pain and disability are discouraged.
2. *Exposure with “control”*: The exposure with control will be directed to activities reported by patients as painful, feared or avoided like: bend the trunk forward to catch an object on the floor or to keep in a sitting position. The patient will be invited to confront such activities.
3. *Lifestyle change*: physical activity and lifestyle advice will include an invitation to gradually increase physical activity levels based on their preference and presentation, advice on sleep hygiene, stress management strategies, and social re-engagement behavioral modification.

### Statistical analysis

All randomized patients will be analyzed on an intention-to-treat basis. Baseline characteristics will be presented by the treatment group. Binary and categorical variables will be summarized by frequencies and percentages. The between-group differences (effects of treatment) and their respective 95% CIs will be calculated by constructing linear mixed models. Treatment effect for the primary and continuous secondary outcomes will be estimated using mixed linear models, considering the correlation within the individual. The mixed linear model will include random intercept adjusted with the baseline score, time as categorical, and the interaction between treatment and time. We will use strategies to reduce the attrition rate during follow-ups as telephone calls and an active search of information.

Two primary outcome variables were considered for the calculation of sample size. For pain intensity, an improvement of at least 1-point on the NPRS (SD=1) and a mean difference of at least 5 points for the ODI score (SD=10). The following specifications were considered: α=5%, 80% statistical power, effect size of 0.27 for *F*-test, and a follow-up attrition rate of 15%. The final sample size will be comprised of 152 patients (76 per group) (G*Power©, University of Dusseldorf, Germany). A moderator effect of baseline variables including the expectation to treatment will be calculated using regression analysis and structural equation modeling (*path analysis*) (AMOS (SPSS, v.22). Data will be coded and entered into the Statistical Package for the Social Sciences (SPSS), version 22 (Chicago, IL, IBM corp., USA) program for analysis and the significance level will be established at 0.05. The Bonferroni correction will be used to adjust the *p*-values for multiple primary outcomes [47]. The data collected will be stored and coded to protect patient confidentiality. In order to handle missing data, we will replace the value with the last observation carried forward. The researchers involved in the study will have access to the final trial dataset and, upon request, it can be shared for the purposes of results conference and publication. The results of the study will be disseminated to interested parties through publications in an appropriate journal.

## Discussion

The pain and disability related to CLBP may compromise many aspects of individual daily life. Non-specific CLBP is best seen as neurobiological and behavioral responses to individuals’ actual and/or perceived threat to their body, lifestyle, or social circumstances and/ or disruption to their homeostasis. In such a scenario, CFT was developed to consider these interacting processes involved in CLBP which demands a flexible multidimensional clinical reasoning framework [9].

Despite its promising approach centered on the biopsychosocial model, there are few RCTs available in the literature [10–12], and the majority are conducted always by the same researcher group, noteworthy are the limitations of the first trials [48, 49]. The RCTs previously published [10–12] showed high attrition rates during follow-up (greater than 24%), failed to follow the intention-to-treat principles, and to blind assessors involved in the follow-up assessments.

To the best of our knowledge, the current study will be the first to compare CFT vs. sham intervention. Sham-controlled RCTs may help to understand the influence of non-specific factors on treatment outcomes. Considering complex interventions as CFT, it is imperative to understand the impact of contextual factors on outcomes.

## Trial status

Protocol: The study was registered prospectively on ClinicalTrials.gov (Protocol Number: NCT04518891). Last Update Posted: June 18, 2021.

Date recruitment began: May 17, 2021

Approximate date when recruitment will be completed: July 17, 2023

## Supplementary Information

**Additional file 1: Supplementary material 1.** Description of the active treatment and sham control of the current study according to TIDieR (Template for Intervention Description and Replication)-Placebo.

## Data Availability

The datasets used and/or analyzed during the current study will be available from the corresponding author on reasonable request.

## Abbreviations

CFT: Cognitive functional therapy
CLBP: Chronic low back pain
RCTs: Randomized clinical trials
NPRS: Numerical pain rating scale
CONSORT: Consolidated Standards of Reporting Trials
SPIRIT: Standard Protocol Items: Recommendations for Interventional Trials
TIDieR: Template for Intervention Description and Replication
MCDID: Minimum clinically important difference
ODI: Oswestry Disability Index
PSEQ: Pain Self-Efficacy Questionnaire
GPE: Global Perceived Effect of improvement with treatment
STES: Standford ExpectationTreatment Scale
PHQ-9: Patient Health Questionnaire 9-item
TKS: The Tampa Scale for Kinesiophobia
PCS: Pain catastrophizing scale
SPSS: Statistical Package for the Social Sciences
SD: Standard deviation
