# Supplementary material for: Efficacy of the cognitive functional therapy (CFT) in patients with chronic nonspecific low back pain: a study protocol for a randomized sham-controlled trial
Source: Trials. 2022 Jul 4;23:544. doi: 10.1186/s13063-022-06466-8 (PMC9252077; doi:10.1186/s13063-022-06466-8)
Supplement: Supplementary file 1 — Additional file 1: Supplementary material 1. Description of the active treatment and sham control of the current study according to TIDieR (Template for Intervention Description and Replication)-Placebo. [file 13063_2022_6466_MOESM1_ESM.docx]

**Supplementary material 1**

**Description of the active treatment and sham control of the current study according to TIDieR (Template for Intervention Description and Replication)-Placebo**

| **Active intervention** | **Placebo/sham intervention** |
| --- | --- |
| **1 Brief Name** |  |
| Provide the name or a phrase that describes the intervention* | Provide the name or a phrase that describes the placebo/sham intervention |
| Cognitive Functional Therapy (CFT) | Sham photobiomodulation + neutral talking |
| **2 Why** |  |
| Describe any rationale, theory, or goal of the elements essential to the intervention | Describe any rationale, theory, or goal of the elements essential to the placebo/sham intervention* |
| Chronic low back pain (CLBP) is a public health problem, and there is strong evidence that it is associated with a complex interaction of biopsychosocial factors. However, a large number of interventions for CLBP are not personalized to encompass specific individualized patient needs. In this sense, the CFT is a promising new intervention that deals with potentially modifiable multidimensional aspects of pain, is a flexible and integrated behavioral approach for individualizing the management of people with disabling CLBP. Despite it’s a promising approach centered in the biopsychosocial model, there are few RCTs available in the literature and to our knowledge, this will be the first study to compare CFT versus sham intervention. | One can argue that for complex interventions such as CFT, the comparison with a sham-procedure is not suitable. Consequently, the challenge is to deliver the same amount of fake therapeutic ingredients and patient-therapist interactions. Patients allocated to this group will receive two interventions: sham photobiomodulation + neutral talking. The photobiomodulation will be adopted in the current study because of the absence of sensory perceptions during the application. The device will be used with the internal cables disconnected, however, it will be possible to handle it and adjust doses and alarms as if they were simulating a real clinical situation as well as to increase the credibility. In addition, a neutral talking control therapy of at least 15 minutes will be provided to patients in each session, to improve the similarity of interventions. |
| **3 What (materials)** |  |
| Describe any physical or informational materials used in the intervention, including those provided to participants or used in intervention delivery or in training of intervention providers. Provide information on where the materials can be accessed (such as online appendix, URL) | Describe any physical or informational materials used in the placebo/sham intervention, including those provided to participants or used in intervention delivery or in training of intervention providers. Provide information on where the materials can be accessed (such as an online appendix, URL) |
| Previous training was carried out by the therapist responsible for administering the CFT intervention, and involved: (1) training of 106 hours of CFT by an experienced CFT tutor and (2) videos of the physiotherapist administering the CFT approach was analyzed by a certified physiotherapist. CFT is a complex behavioral approach that does not require physical materials. All the patients will receive a booklet with information regarding low back pain and advice on strategies of self-management (adapted: Jenkins HJ, Moloney NA, French SD, et al. Using behaviour change theory and preliminary testing to develop an implementation intervention to reduce imaging for low back pain. BMC Health Serv Res. 2018 Sep 24;18(1):734). | Previous training for neutral talking control therapy, without containing any educational component on pain. Patients will be treated with detuned photobiomodulation device (we will use 904Nm Ibramed Infrared – no-visible beam), without any emission of therapeutic dose (0J). All the patients will receive a booklet with information regarding low back pain and advice on strategies of self-management (adapted: Jenkins HJ, Moloney NA, French SD, et al. Using behaviour change theory and preliminary testing to develop an implementation intervention to reduce imaging for low back pain. BMC Health Serv Res. 2018 Sep 24;18(1):734). |
| **4 What (procedures)** |  |
| Describe each of the procedures, activities, and/or processes used in the intervention, including any enabling or support activities | Describe each of the procedures, activities, and/or processes used in the placebo/sham intervention, including any enabling or support activities |
| The CFT intervention has three broad components:  1. Making sense of pain: a reflective process that combines the person’s own narrative (interview) and experience to develop a customized relevant, multidimensional understanding of pain for the patient. In this process, maladaptive beliefs related to vicious cycle of persistent pain and disability are discouraged.  2. Exposure with ‘control’: The exposure with control will be directed to activities reported by patients as painful, feared or avoided like: bend the trunk forward to catch an object on the floor or to keep in a sitting position. The patient will be invited to confront such activities.  3. Lifestyle change: physical activity and lifestyle advice will include invitation to gradually increasing physical activity levels based on their preference and presentation, advice on sleep hygiene, stress management strategies and social re-engagement behavioral modification. | The points for applying fake stimulation will be in nine sites, applicate on the patient’s lumbar region: three central sites on top of the spinous processes (between T11 and T12, L2 and L3, L5 and S1); in the same direction, but laterally, three sites on the left and three on the right (on the paravertebral muscles), totalizing up 27 minutes. A neutral talking control therapy of at least 15 minutes will be provided to patients in each session. Maladaptive beliefs will not be challenged; however, the therapists will be trained to show interest and warmth, empathy and encouraging participants to discuss neutral topics such as hobbies, sports, and current affairs. No advice or problem solving will be given, and any attempt to talk about emotional issues will be kindly discouraged and the talking will be redirect to neutral tropics. |
| **5 Who provided** |  |
| For each category of intervention provider (such as psychologist, nursing assistant), describe their expertise, background, and any specific training given | For each category of placebo/sham intervention provider (such as psychologist, nursing assistant), describe their expertise, background, and any specific training given |
| A physiotherapist with 5 years of clinical experience (first author), who performed a 106-hour pre-training with a CFT expert. | Same as active intervention. |
| **6 How** |  |
| Describe the modes of delivery (such as face to face or by some other mechanism, such as internet or telephone) of the intervention and whether it was provided individually or in a group | Describe the modes of delivery (such as face to face or by some other mechanism, such as internet or telephone) of the i placebo/sham intervention and whether it was provided individually or in a group |
| Intervention was delivered face to face and individually. | Same as active intervention. |
| **7 Where** |  |
| Describe the type(s) of location(s) where the intervention occurred, including any necessary infrastructure or relevant features | Describe the type(s) of locations(s) and settings where the placebo/sham intervention occurred, including any necessary infrastructure or relevant features |
| The intervention occurred in two places: (I) physiotherapy outpatient clinic in a basic health unit in Ribeirão Preto/SP, Brazil, through patients referred for physiotherapy; and (II) University of São Paulo (laboratory), campus of Ribeirão Preto/SP, Brazil, through open invitations to the community. | Same as active intervention. |
| **8 When and how much** |  |
| Describe the number of times the intervention was delivered and over what period of time including the number of sessions, their schedule, and their duration, intensity, or dose | Describe the number of times the placebo/sham intervention was delivered and over what period of time including the number of sessions, their schedule, and their duration, intensity, or dose. If relevant, include the duration of the pre-, and post-randomisation consultations |
| The experimental group will receive individualized CFT in a pragmatic manner (5 to 7 sessions, once a week) based on the clinical progression of participants. We plan to recruit the sample between 2021 to 2023. | Patients in the sham intervention group will receive 6 sessions, lasting 45 minutes, once a week. We plan to recruit the sample between 2021 to 2023. |
| **9 Tailoring** |  |
| If the intervention was planned to be personalised, titrated or adapted, then describe what, why, when, and how | If the placebo/sham intervention was planned to be personalised, titrated or adapted, then describe what, why, when, and how |
| A customized progressive self-management program will be provided tailored to the individual’s valued goals, directed at changing cognitive, movement and lifestyle behaviors considered provocative or aggravating of their condition. | Sham intervention will be standardized for all volunteers. |
| **10 Modifications** |  |
| If the intervention was modified during the course of the study, describe the changes (what, why, when, and how) | If the placebo/sham intervention was modified during the course of the study, describe the changes (what, why, when, and how) |
| Not applicable. | Not applicable. |
| **11 How well: planned** |  |
| Planned: If intervention adherence or fidelity was assessed, describe how and by whom, and if any strategies were used to maintain or improve fidelity, describe them | Planned: If placebo/sham intervention adherence or fidelity was assessed, describe how and by whom, and if any strategies were used to maintain or improve fidelity, describe them |
| The physiotherapist responsible for the active intervention was previously trained, in addition, as a strategy to control for the treatment fidelity on both arms of the study, we will adopt the framework developed by the NIH Behavior Change Consortium (BCC). To control for the “Intervention Delivery”, the time of therapist-patient interaction, number of sessions and the different components of the intervention administered, as well as video recordings along the trial will be registered. Also, regular meetings to discuss the clinical cases will be performed. Furthermore, the “Intervention receipt” will be assessed in two different manners: i) on the completion of the study, each participant will be submitted to a manipulation check and they will be asked about the group they think they were at and ii) patients in both groups will be invited to summarize at the beginning of each session how the treatment impact their lives. | Same as active intervention. |
| **12 How well: actual** |  |
| Actual: If intervention adherence or fidelity was assessed, describe the extent to which the intervention was delivered as planned | Actual: If placebo/sham intervention adherence or fidelity was assessed, describe the extent to which the intervention was delivered as planned |
| Not applicable. | Not applicable. |
| **13 Measuring the Success of Blinding** | |
| Was blinding measured, and if so: how, and what were the results of such measurement? | |
| Despite it is a sham-controlled RCT, considering the differences in the interventions administered in both study arms, it will not be possible to blind the therapist, however, patients will be blinded to sham procedure. The assessor and the participants will not have access to what type of treatment the participant will be given. After the end of the interventions, all volunteers will be asked which treatment group they believe they were allocated to, with the following response options: (I) Placebo; (II) Active; (III) I don't know. | |
